# Characterization of a large cluster of HIV-1 A1 infections detected in Portugal and connected to several Western European countries

**DOI:** 10.1038/s41598-019-43420-2

**Published:** 2019-05-10

**Authors:** Pedro M. M. Araújo, Alexandre Carvalho, Marta Pingarilho, BEST-HOPE study group, Domítilia Faria, Raquel Pinho, José Ferreira, Paula Proença, Sofia Nunes, Margarida Mouro, Eugénio Teófilo, Sofia Pinheiro, Fernando Maltez, Maria José Manata, Isabel Germano, Joana Simões, Olga Costa, Rita Corte-Real, António Diniz, Margarida Serrado, Luís Caldeira, Nuno Janeiro, Guilhermina Gaião, José M. Cristino, Kamal Mansinho, Teresa Baptista, Perpétua Gomes, Isabel Diogo, Rosário Serrão, Carmela Pinheiro, Carmo Koch, Fátima Monteiro, Maria J. Gonçalves, Rui Sarmento e Castro, Helena Ramos, Joaquim Oliveira, José Saraiva da Cunha, Vanda Mota, Fernando Rodrigues, Raquel Tavares, Ana Rita Silva, Fausto Roxo, Maria Saudade Ivo, José Poças, Bianca Ascenção, Patrícia Pacheco, Micaela Caixeiro, Nuno Marques, Maria J. Aleixo, Telo Faria, Elisabete Gomes da Silva, Ricardo Correia de Abreu, Isabel Neves, Ana B. Abecasis, Nuno S. Osório

**Affiliations:** 1 Life and Health Sciences Research Institute (ICVS), School of Medicine, University of Minho, Braga, Portugal; 2 ICVS/3B’s - PT Government Associate Laboratory, Braga, Guimarães, Portugal; 3 Hospital de Braga, Braga, Portugal; 4 Global Health and Tropical Medicine-GHTM, Institute for Hygiene and Tropical Medicine, Universidade NOVA de Lisboa, UNL, Lisbon, Portugal; 5 Centro Hospitalar do Algarve (CHA), Hospital de Portimão, Portimão, Portugal; 6 CHA, Hospital de Faro, Faro, Portugal; 7 Centro Hospitalar Baixo Vouga, Aveiro, Portugal; 8 Centro Hospitalar Lisboa Central (CHLC), Hospital Capuchos, Lisboa, Portugal; 9 CHLC, Hospital Curry Cabral, Lisboa, Portugal; 10 CHLC, Hospital São José, Lisboa, Portugal; 11 CHLC, Lab, Lisboa, Portugal; 12 Centro Hospitalar Lisboa Norte (CHLN), Hospital Pulido Valente, Lisboa, Portugal; 13 CHLN Hospital de Sta Maria, Lisboa, Portugal; 14 CHLN, Lab, Lisboa, Portugal; 15 Centro Hospitalar Lisboa Ocidental (CHLO), Hospital Egas Moniz, Lisboa, Portugal; 16 CHLO, Lab, Lisboa, Portugal; 17 Centro Hospitalar do Porto (CHP), Porto, Portugal; 18 CHP, Lab, Porto, Portugal; 19 Centro Hospitalar de São João (CHSJ), Porto, Portugal; 20 CHSJ, Lab, Porto, Portugal; 21 Centro Hospitalar das Universidades de Coimbra (CHUC), Coimbra, Portugal; 22 CHUC, Lab, Coimbra, Portugal; 23 Hospital Beatriz-Ângelo, Loures, Portugal; 24 Hospital de Santarém, Santarém, Portugal; 25 Hospital de São Bernardo, Setúbal, Portugal; 26 Hospital Fernando da Fonseca, Amadora, Portugal; 27 Hospital Garcia da Orta, Almada, Portugal; 28 ULS Baixo Alentejo, Beja, Portugal; 29 ULS, Matosinhos, Portugal

**Keywords:** Bioinformatics, HIV infections

## Abstract

HIV-1 subtypes associate with differences in transmission and disease progression. Thus, the existence of geographic hotspots of subtype diversity deepens the complexity of HIV-1/AIDS control. The already high subtype diversity in Portugal seems to be increasing due to infections with sub-subtype A1 virus. We performed phylogenetic analysis of 65 A1 sequences newly obtained from 14 Portuguese hospitals and 425 closely related database sequences. 80% of the A1 Portuguese isolates gathered in a main phylogenetic clade (MA1). Six transmission clusters were identified in MA1, encompassing isolates from Portugal, Spain, France, and United Kingdom. The most common transmission route identified was men who have sex with men. The origin of the MA1 was linked to Greece, with the first introduction to Portugal dating back to 1996 (95% HPD: 1993.6–1999.2). Individuals infected with MA1 virus revealed lower viral loads and higher CD4^+^ T-cell counts in comparison with those infected by subtype B. The expanding A1 clusters in Portugal are connected to other European countries and share a recent common ancestor with the Greek A1 outbreak. The recent expansion of this HIV-1 subtype might be related to a slower disease progression leading to a population level delay in its diagnostic.

## Introduction

The Human immunodeficiency virus 1 (HIV-1) pandemic is characterized by an extensive genetic diversity of the pathogen, a consequence of high viral replication, recombination, and mutation rates^1,2^. Among the four existing HIV-1 groups^3^ only the M group has a global distribution. This group is consensually divided into several subtypes (A-D, F-H, J, K), sub-subtypes (A1 to A4 and F1 and F2) and an increasing number of recombinant forms^4,5^. The genetic diversity among HIV-1 subtypes may cause different disease progression rates^6–9^, advantages in specific transmission routes^10,11^, different capacities to evade the immune response^12,13^ and was associated with specific differential therapy outcomes^14–17^. Moreover, HIV-1 subtypes surveillance proved to be an important tool to reconstruct the history of an epidemic over time^18,19^.

The global distribution of HIV-1 subtypes has been heterogeneous but well compartmentalized^20^. However, in recent decades the HIV-1 subtype geographic distribution pattern became more complex^21–23^. The number of non-B infections is increasing^24–30^ in Western and Central Europe (WCE), where subtype B has long been predominant. The infections by non-B subtypes in this region were commonly linked to immigrants from other geographic locations^19,21^. Nevertheless, the transmission of previously rare subtypes is increasing among native individuals^25,31–35^.

Portugal has one of the highest numbers of new HIV infections among Western European countries and an atypical HIV-1 subtype diversity due to the high prevalence of G subtype^25,27,36^. When studying new HIV-1 infections in Europe from 2002 to 2005, Abecasis *et al*. highlighted Portugal as the European country with more non-B subtype infections (60.8%)^19^. A recent study^25^ of a Portuguese cohort from the region of Minho (Northwest) reported the predominance of G and B subtypes followed by recombinant forms. The authors also showed the existence of non-B and non-G transmission clusters, related to the increasing number of new infections by A1 and F1 sub-subtypes^25^.

In the present study, we performed a multicenter characterization of the HIV-1 sub-subtype A1 infections in Portugal. Using a phylogenetic approach, we inferred regional and multinational transmission chains while estimating their geographic and temporal origins.

## Results

### Characterization of the phylogenetic relationship among study sequences

**Figure 1:**
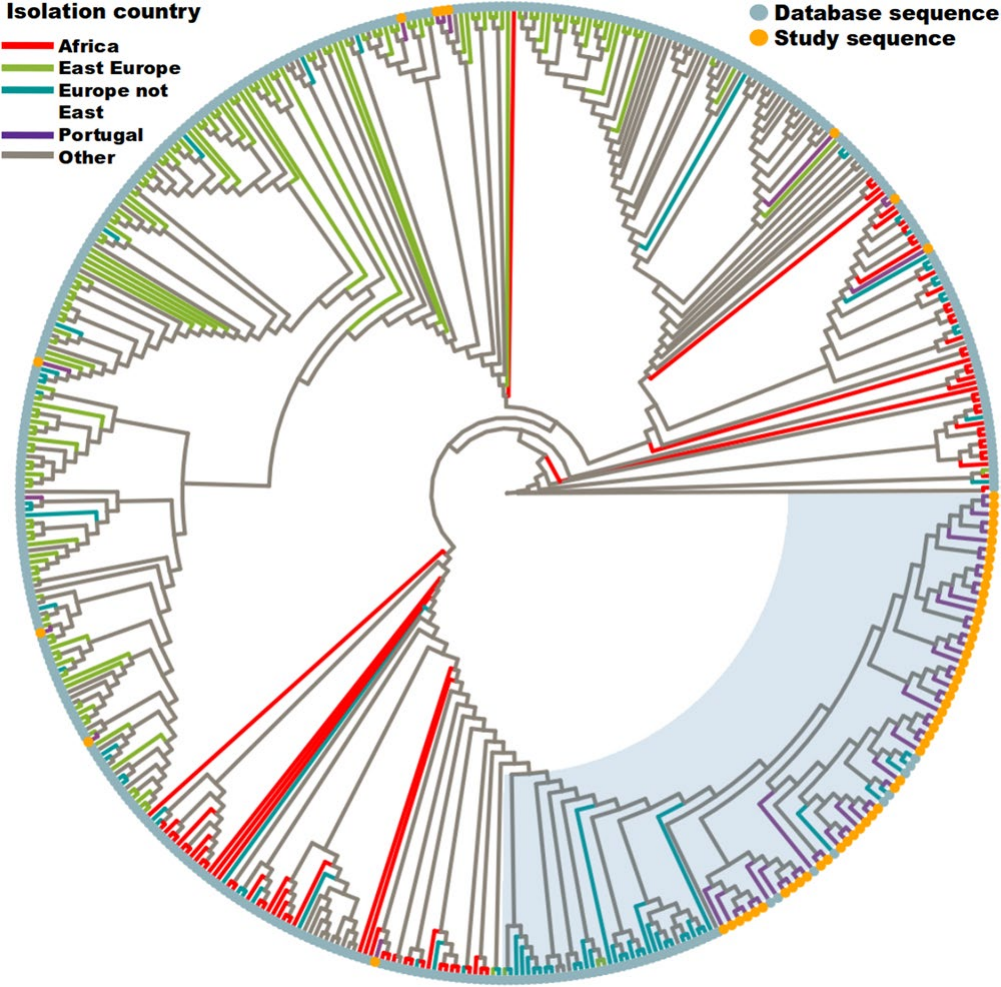
Phylogenetic representation of the A1 sequences isolated in Portugal and closely related sequences from databases. Circular cladogram representation of the Maximum likelihood tree (Supplementary Fig. 1) with the study A1 sequences and the closely related database sequences (n = 490). Branch colours indicate the geographical origin of the sequences. The tip points indicate if the taxa are an A1 sequence from this study or a database sequence. Most of this study sequences cluster together in a well-defined area of the phylogeny (52 of 65), here further mentioned as the main A1 cluster (MA1). The light grey background encompasses the MA1 and closely related sequences used for further analyses (n = 99, aLRT SH-like branch support = 0.95).

The phylogenetic representation (Fig. 1) demonstrated that 80% (52 of 65) of the A1 sub-subtype virus isolated in 14 Portuguese hospitals clustered together in a main A1 clade (MA1, n = 61). Most of the study sequences (96%, 50 of 52) within MA1 were isolated from individuals of Portuguese nationality. MA1 also included sequences obtained from public databases isolated in Spain, France, and United Kingdom (10%, 5 of 61). MA1 was nested in the phylogeny with most of the sequences isolated in WCE.

The remaining sub-subtype A1 isolates sequenced in this study (13 of 65) are scattered over the tree. From which, five clustered with sequences isolated in African countries. Among those are four sequences isolated from African immigrants (Supplementary Table 1). The other eight, including three sequences isolated from Ukrainian nationals, were highly related to isolates from East Europe (see Supplementary Table 1).

Overall, these results suggest that there were several introductions of sub-subtype A1 viruses in Portugal, from at least three different geographic regions: Europe not East; Africa; and Eastern Europe. However, most of the sampled infections (>80%) were caused by a monophyletic introduction of a sub-subtype A1 virus.

### Most recent common ancestor characterization

**Figure 2:**
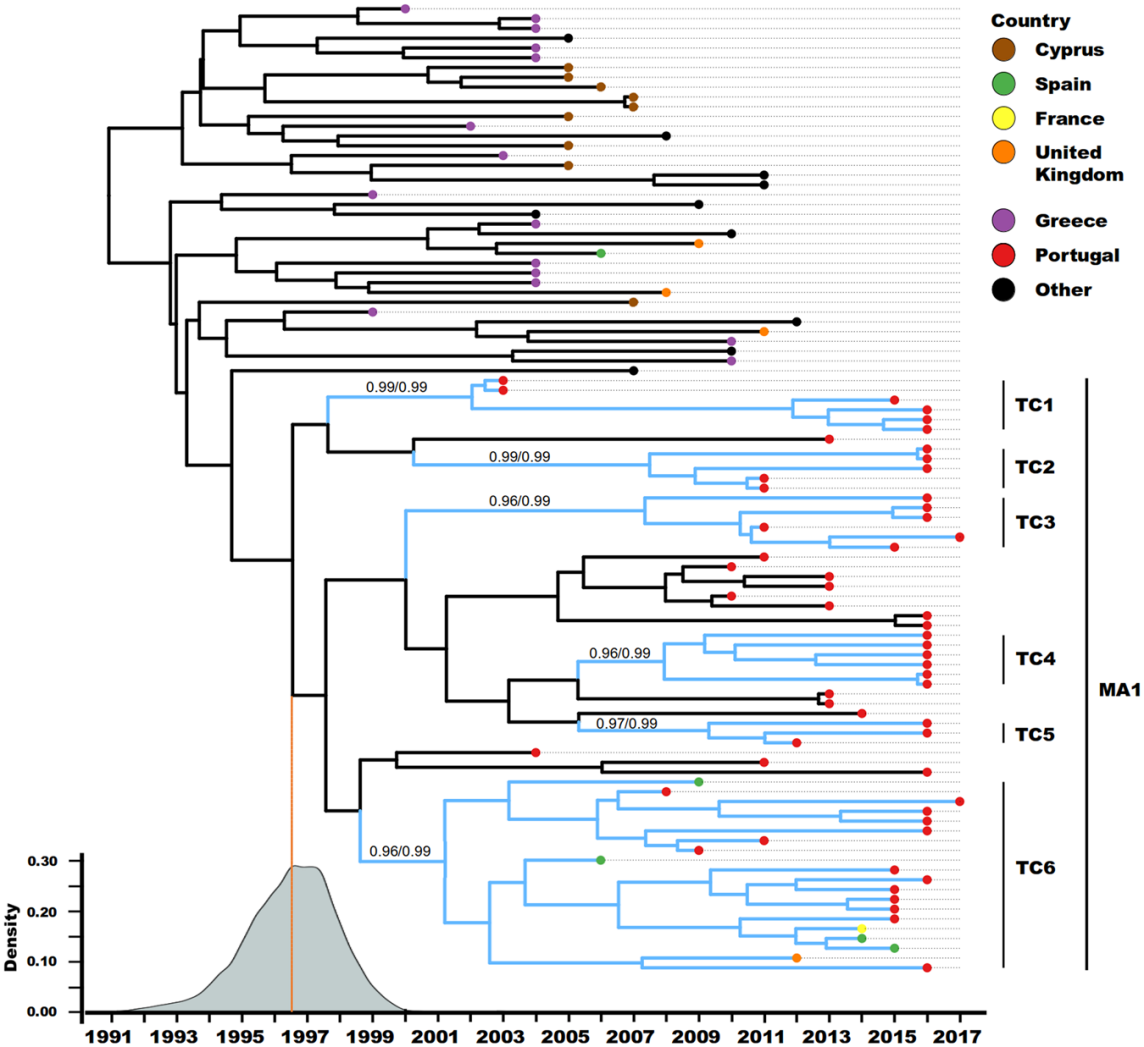
Evolution of the study sub-subtype A1 transmission chain through its most recent history in several European countries. Maximum clade credibility tree summarised from the output trees from the BEAST analyses (n = 99). The tip point colours represent the country where the samples were collected. The blue branch lines indicate that the respective HIV-1 sequence belong to a transmission cluster. The vertical black bars demonstrate the taxa included in each transmission cluster and the main A1 clade (MA1). The aLRT SH-like branch support and posterior probability (pp) of each of the transmission clusters is displayed (aLRT/pp). The density plot in the bottom left refers to the time of the most recent common ancestor of the MA1. The country of sampling of the taxa labelled as others is: USA (3); Belgium (3); Sweden (1); Australia (1); The Netherlands (1), and Kuwait (1).

To better understand the most recent history of the sub-subtype A1 spread in Portugal the MA1 and closely related database sequences (total n = 99) were analyzed. This dataset is mainly composed by isolates from Western (France, Portugal, Spain, and United Kingdom) and Southern (Cyprus and Greece) European countries (Fig. 2). The estimate for the date of the most recent common ancestor (MRCA) of the MA1 is mid 1996 (95% highest posterior density (HPD): 1993.6–1999.2). The taxa outside the MA1 but closely related to it, sharing and older MRCA (1990.9; 95 HPD: 1986.8–1994.6), were almost exclusively sampled in Greece and Cyprus. Therefore, this data demonstrates the European ancestry of the MA1 viruses expanding in Portugal.

### Transmission clusters characterization

From the 61 taxa in the MA1, 46 (~75%) are part of six well-delimited transmission clusters (TC1 to TC6, Table 1). TC5 is the smallest with 3 sequences, followed by TC2 with 5, TC1, TC3 and TC4 with 6. Interestingly, TC6 included 20 sequences. Eight sequences in all the clusters lacked demographic information. Among those with available data, two sequences were isolated from females and 36 from males. The self-reported transmission route for all transmission clusters is 100% sexual, being the majority (59%) men who have sex with men (MSM). However, in TC5 and TC2 self-reported heterosexual transmission dominated, while in TC4 only MSM is reported. These results suggest a strong association of the MA1 transmission clusters with males and MSM route of transmission.

**Table 1:** Characterization of the six transmission clusters identified in the main A1 cluster.

Cluster	No of individuals				Route of transmission (n)	Sampling date		Time of MRCA		Cluster Depth	Sample origin
	Total	Male	Female	Unknown		Mean	Range	Mean	95% HPD		
TC1	6	4	0	2	MSM (3), Heterosexual (1), Unknown (2)	2011.7	2003–2016	2002	2000.8–2002.9	14	Multicenter^a^
TC2	5	3	2	0	MSM (1), Heterosexual (4)	2014	2011–2016	2007.5	2004.9–2009.8	8.5	Multicenter^a^
TC3	6	6	0	0	MSM (4), Heterosexual (2)	2015.2	2011–2017	2007.3	2004.2–2010.0	9.6	Multicenter^a^
TC4	6	6	0	0	MSM (6)	2016	2016–2016	2007.9	2005.0–2010.7	8.1	Multicenter^a^
TC5	3	3	0	0	MSM (1), Heterosexual (2)	2014.7	2012–2016	2009.3	2006.9–2011.4	6.7	Multicenter^a^
TC6	20	14	0	6	MSM (12), Heterosexual (2), Unknown (6)	2013.5	2006–2017	2001.2	1998.8–2003.2	15–8	Multicenter^a^, Multinational^b^

^a^Samples from more than one of the 14 Portuguese hospital centers.

^b^France, Portugal, Spain, and United Kingdom.

All the transmission clusters (TC) in this study are multicenter and TC6 is multinational. Three transmission clusters (2, 3, and 5) are composed by isolates from the cities of Braga and Porto, in the northern region of Portugal. The same pattern is observed for the central region of Portugal, with isolates from Aveiro, Coimbra, Lisbon and Setúbal in TC1 and TC4. This result suggests some level of regional compartmentalization in the transmission of MA1 viruses in Portugal. However, TC6, with 20 taxa, includes isolates from several Portuguese regions (North: Braga, Centre: Lisbon and Setúbal; South: Faro) and isolates from France, Spain, and the United Kingdom. This supports that the transmission history of this cluster is not restricted to Portugal.

The estimates for the time of the MRCA for each cluster were at the first two decades of the 2000s. The cluster depth (diagnostic date of the most recent sample minus the time of MRCA) showed that transmission of TC 1, 3, and 6 are expected to be ongoing for at least one decade. It is also of notice that all the transmission clusters have at least one taxon isolated in the years of 2016 or 2017, suggesting the emergent character of these transmission clusters.

### Phylogeographic root and discrete rates among countries

**Figure 3:**
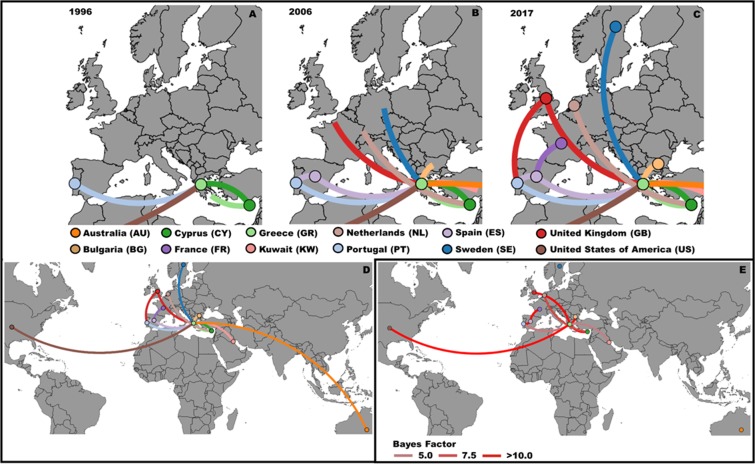
Phylogeographic evolution of the Main A1 clade. Geographical display of the phylogeny built using BEAST with the country of the sequence origin as discrete trait. (**A**) In the year of 1996 this transmission history reached Western Europe, arriving to Portugal. (**B**) After a decade, in 2006, viruses from this clade are expected to be found in several countries from different geographic regions. Some countries, like Portugal, started now being donors and not only receivers in this transmission history. (**C**) In 2017 the spread among several European countries is established. (**D**) Global representation of this transmission history; (E) Rates between country pairs with Bayes Factor superior to 5.

A phylogeographic analysis was performed using the country of sample collection as a discrete trait (Fig. 3, Supplementary Video). Greece had the highest posterior probability for the tree root (pp = 0.84). Nevertheless, most of the countries in the analysis are in the 95% HPD interval for the tree root. A total of 6 (ES-PT, CY-GR, GB-GR, GR-US, ES-FR, BG-GR) pairwise rates of diffusion between locations have a strong support value (Bayes factor >10^37^, Fig. 3E, Supplementary Table 2). Greece is the most common location among well supported rates of diffusion, being present in four. These rates highlight that several geographic locations, at a given point of the transmission history were not only receivers but also donors.

### CD4^+^ T-cell levels and amino acid variant characterization

**Figure 4:**
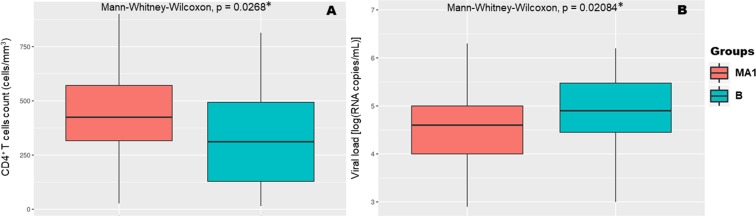
Comparison of infection progression outcomes between the MA1 study group and the control group (B subtype). There were statistically significant differences between the two study groups regarding CD4^+^ T-cell counts (**A**) and viral load (**B**). *At a significance level of 0.05 using the Mann-Whitney-Wilcoxon Test (p < 0.05).

To investigate the association of MA1 viruses with CD4^+^ T-cell counts or viral load a comparison with a matched control group was performed. The groups are composed by individuals infected with MA1 viruses (n = 50, 11 cases were excluded due to lack of clinical data) and subtype B (n = 42), matching for gender, age and proportion of ambiguous sites (PAS - as a surrogate of time of infection^38–41^). The values for the CD4^+^ T-cell counts and viral load of the two groups are statistically different (p < 0.05, Mann-Whitney-Wilcoxon Test, Fig. 4A,B). To investigate if the viral genotype could contribute to these findings the prevalence of the existing amino acid variants was compared between the two groups. For 15 amino acid sites in the protease and 19 in the reverse transcriptase there are marked differences (Bonferroni correction of Fisher Exact Test, p < 0.05) in the proportion of the variants when comparing MA1 vs. B (Supplementary Tables 3 and 4). Interestingly, some of these sites (protease: 14, 20, 35, 36, 37, 41, 57, 63, 64, 71, and 89; reverse transcriptase: 207) were previously reported to influence viral fitness (Supplementary Tables 3 and 4)^42–46^. We evaluated if there were associations between the amino acid variants and CD4^+^ T-cell counts or viral load. The results were not statistically significant. Of note, the amino acid sites 14, 36, 71 and 89 in the protease and the site 207 in the reverse transcriptase were associated with differences in the viral load and CD4^+^ T-cell counts before adjustment for multiple comparisons (Supplementary Tables 5 and 6).

## Discussion

Our results demonstrate that the sub-subtype A1 viruses circulating in Portugal had three distinct historical and geographic origins: related to the Eastern European outbreak; isolates from the African continent; and the emergent Greek-Cyprus epidemic. A similar pattern was observed by Lai A. *et al*.^30^ when characterizing the sub-subtype A1 epidemic in Italy. These authors concluded that sub-subtype A1 entered Italy originated from three geographic locations^30^. We found no evidence of an evolutionary relationship between the A1 sequences from that study^30^ and the sequences herein reported. Thus, the introductions of sub-subtype A1 viruses in Portugal and Italy are likely to be independent.

Only a minority of A1 sequences isolated in Portugal was found in migrants and clustered with sequences isolated from their countries of origin. These cases are likely to be infections acquired abroad and with limited spread in Portugal. In contrast, the Greece-Cyprus introduction resulted in a large transmission chain established among Portuguese natives, the MA1. The sub-subtype A1 has been increasing in prevalence in Greece^47^, representing 30% of the new diagnosis between 2002 and 2005^19^. This expansion continued^26,48^ making sub-subtype A1 responsible for most of HIV-1 infections in some Greek cohorts, suggesting a possible advantage to previous established subtypes. The high prevalence of sub-subtype A1 in Greece may have been the preponderant factor for the establishment of the link between Portugal and Greece suggested by our analyses. Therefore, our results highlight an additional case of increasing number of infections by a non-B subtype in an European country unrelated with migrants from outside Europe.

The characterization of the six transmission clusters made evident that the main clade of sub-subtype A1 infections in Portugal is related to MSM route of transmission among Portuguese. Moreover, this characterization revealed that there was some level of compartmentalization of the HIV-1 transmission in Portugal at a region level. However, TC6 encompassed sequences isolated in several countries, indicating a larger geographical spread. The disparity seen in TC5, composed only by males but mainly reporting heterosexual transmission route can be explained by the previously described MSM under-reporting^49,50^. Furthermore, “missing-links” can still exist, related to unavailable data that could help explain this disparity. Except for TC4, all the transmission clusters have sequences isolated from a range of years superior to six. This shows that our approach was successful in characterizing the most recent history of these clusters. Our previous work studying HIV-1 infections from all subtypes in the region of Minho (Portugal) from the years 2000 to 2012^25^ showed a statistical significant increase in the detection of A1 infections. In the present work we characterized a monophyletic clade (MA1) with six transmission clusters, an MRCA not older than 2000 and less than 16 years of cluster depth. Together these results demonstrate that the transmission history of the sub-subtype A1 in Portugal occurred mainly in the last two decades.

We believe that even if older samples were available the dating of the MRCA of the MA1 as 1996 and the beginning of the transmission clusters in the 2000s would not change significantly. These estimations are in accordance to what was previously described by Carvalho A. *et al*.^25^ about an A1 transmission chain, and the report by Esteves A. *et al*.^51^ of sub-subtype A1 virus circulation in Portugal in the late 90s (1997 to 2001).

The indication of Greece as the geographic location of origin for the spread of these sub-subtype A1 viruses in other European countries highly depends on the data available in public databases. However, our aim was to characterise the most recent history of these A1 sequences in Europe. We cannot exclude the possibility of missing data being brought to light showing that there were intermediate steps in the transmission not visible in our analyses.

Upon introduction of a HIV-1 subtype to a novel region its capacity to become established and lead to the formation of large transmission chains is somewhat elusive. The sub-subtype A1 was previously characterised as a viral genotype that could lead to a slow disease progression in different host populations^7–9,52–55^. Here we observed a statistically significant difference between the CD4^+^ T-cell counts, and viral load of patients followed in Portugal and infected by virus of the MA1 compared to subtype B. Given that the two groups were normalised for factors known to influence CD4^+^ T-cell counts and viral load, these results suggest that the infection by MA1 viruses may lead to a lower viral load and a slower decrease of the CD4^+^ T-cell count when compared with infections by a virus of the predominant subtype.

A total of 24 sites, 15 in protease and 19 in reverse transcriptase, showed statistically significant differences in the amino acid proportions between subtype groups. Of those sites, 12 were previously associated with changes in the viral fitness (Supplementary Tables 3 and 4). When comparing several protease mutations, M. Parera *et* *al*.^42^ demonstrated that modifications in sites 14, 20, 35, 36, 37, 41, 57, 63, 64, 71 and 89 had an impact in this protein catalytic efficiency. The substitution M36I was also reported as having an impact in the virion maturation^43^. The protease site 63 had been associated with compensatory mutations with replication benefits^44^. Variations in the amino acid at protease position 64 were previously associated with differences in the viral replication capacity^45^. The reverse transcriptase site 207 has been associated with viral fitness alterations^46^. Nevertheless, in our study we found no statistically significant association between these sites and viral load or CD4^+^ T-cell counts. To our knowledge, the remaining 12 sites were not previously associated with impact in the viral fitness. We cannot conclude that changes in these sites can influence the natural history of HIV-1 infection, further studies need to be conducted. Moreover, sites that we did not explore, like the C2V3 sequence in HIV-1 envelope^56^, may have a strong impact in the CD4^+^ T-cell counts that we cannot evaluate in the present study. Nonetheless, given the previous reports of a slower CD4^+^ T-cells depletion caused by sub-subtype A1 virus^7–9,52–55^ a better understanding of the genetic characteristics of this HIV-1 sub-subtype is necessary. With our characterization, we provide the basis for a more focused work tackling the clinical significance of sub-subtype A1 genetic diversity and its impact on the European HIV-1 epidemic.

The prevalence of HIV-1 subtypes is changing^21–23^. We need to better understand the causes behind these changes, and their impact on the HIV-1 infection. In recent years sub-subtype A1 became extremely common in Greece^26,48^. Now it is increasing among new infections in Portugal, and potentially other Western European countries. Further HIV-1 surveillance studies are required to evaluate this phenomenon and elucidate its consequences.

## Methods

### Study Population

The inclusion criteria for this study were: (i) availability of partial HIV-1 genome sequence; (ii) infection with sub-subtype B or A1 virus; (iii) absence of previous antiretroviral treatment upon sampling. Data was collected from HIV-1 infected patients followed at Hospital de Braga from 2007 until 2017 and from other 13 Portuguese hospital centres taking part in the BEST HOPE surveillance study from 2016 until 2017 (Supplementary Table 7). For all cases matching the criteria the following patient secondary data was collected: self-reported transmission route; gender; birth year; nationality; self-reported country of infection; date of the viral sample collection for sequencing; CD4^+^ T-cell count at sampling; viral load at sampling. The collection of the patient’s data was performed anonymously after approval by the ethic comities of each healthcare institution.

### Sequencing

Viral RNA was extracted from plasma using MagNA Pure total nucleic acid isolation kits (Roche Applied Science). DNA sequencing, from the reverse transcriptase PCR amplicons was performed with the Trugene^TM^ HIV-1 genotyping system (Siemens Healthcare Diagnostics) and ViroSeq^TM^ genotyping system (Abbott Molecular). The sequenced portion corresponds to the pol region, from position 2253 to 3554 (with some variability related to the used primers). The HIV-1 positions in this study refer to the HXB2 HIV-1 reference genome (GenBank: K03455.1). All multiple sequence alignments were performed using MUSCLE v3.8^57^. The sequences obtained in this study were made available in GenBank (accession numbers in Supplementary Table 8) or are part of the BEST-HOPE program database.

### Database queries

Two databases were queried: the NCBI full nucleotide collection and the HIV reference sequence database (http://www.hiv.lanl.gov/) using BLAST^58,59^. From each of 65 A1 sequences obtained in this study three different input queries were constructed: (i) full sequence; (ii) protease region; (iii) reverse transcriptase region. Each database query generated 10 outputs. We excluded duplicates, results missing collection date or geographic origin, and sequences showing evidence of recombination^60^. Applying these criteria 425 database sequences were selected for this study. Codons related to major antiretroviral drug resistance^61^ were excluded from the multiple sequence alignments prior to phylogenetic analysis.

### Phylogenetic analysis

An alignment of 490 sequences was used to make a phylogenetic reconstruction using PhyML v3.0^62^. The best fitting substitution model was GTR + G4 + I, determined by PhyML SMS^63^. Tree search started from 10 random trees using SPR and NNI methods. The tree with the best likelihood value was selected. For the in-detail study of the most recent history of the sub-subtype A1 in Portugal the main A1 clade, composed by 52 of the study sequences (52 of 65, 80%) and by the most closely related database sequences (total n = 99) was further explored by the following analysis. The maximum likelihood tree was built with the parameters described above. This phylogenetic representation was used to infer the correlation between genetic distance and time, using TempEst v1.5^64^, and to characterize the transmission clusters. The same phylogeny was inferred for a near full-length alignment (for those sequences with available data) to compare topological alterations (Supplementary Fig. 2). Bayesian evolutionary and phylogeographic analyses were performed using BEAST v1.8^65,66^, with BEAGLE library v2.1^67^. The site model used in all the BEAST analyses was GTR + G4 + I for two different codon partitions (1 + 2, 3). According to path and stepping-stone sampling^68–70^, the coalescent skygrid model with an uncorrelated relaxed clock showed the best fit (Supplementary Table 9). Three different runs (random seeds), of 30 million generations, converged to similar values. Outputs were analyzed with Tracer v1.6 to ensure all parameters had an effective sampling size (ESS) superior to 300. The three multiple tree output files were combined, using LogCombiner v1.8^65^ and used to build the maximum clade credibility tree with mean heights using TreeAnnotator v1.8^65^, excluding 10% burn-in. The skygrid plot was also created (Supplementary Fig. 3). In the phylogeographic analysis the sampling country was used as a discrete trait^71^, with a total of 12 different discrete locations (Fig. 3).

### Definition of transmission cluster and tree visualization

The criteria for the definition of a clade as a transmission cluster, chosen after performing a sensitivity analysis of the relevant factors (Supplementary Table 10), were: likelihood ratio test (aLRT) SH-like branch support ≥0.95 (estimated with PhyML v3); branch posterior probability ≥0.99 (estimated with BEAST v1.8); mean cluster genetic distance <0.003 substitutions per site; and maximum genetic distance <0.05 substitutions per site. MEGA v7.0^72^ was used for genetic distance calculation.

For the visualization and manipulation of the trees in this study the software FigTree v1.4 and the R packages ggtree v1.10.5^73^ and APE v5.0^74^ were used. The phylogeographic representation was created with SpreaD3^75^. The countries locations were plotted as their geographic center.

### Statistical analysis

To investigate associations between the infection with the most transmitted sub-subtype A1 virus clade in Portugal (identified as MA1) and the levels of CD4^+^ T-cells and viral load two groups were compared: i) cases infected with sub-subtype A1 virus from MA1 (n = 50, 11 cases were excluded due to lack of clinical data); ii) cases infected with the geographically most prevalent subtype (B) virus (n = 42). All corresponding to treatment naive individuals. The B group was created from the 170 available subtype B cases (Supplementary Table 11) to match the MA1 group in factors known to influence the disease progression: gender (p > 0.05, Fisher’s exact test), age (p > 0.05, Mann-Whitney-Wilcoxon Test), and proportion of ambiguous sites (PAS) (p > 0.05, Mann-Whitney-Wilcoxon Test). PAS was used to normalize the time after infection as it was shown to positively correlate with this variable^38–41^.

To study the association of amino acid variants with these two groups, their prevalence was compared using Fisher’s exact test, at a 0.05 significance level. To infer the association of amino acid variants with changes in the CD4^+^ T-cell counts or viral load, the sequences from the two previously mentioned groups (MA1 and B, n = 92) were grouped according to the amino acid variant present in each site. The groups for each variant for each amino acid position were compared (Mann-Whitney-Wilcoxon Test and T-test, at a 0.05 significance level, using Bonferroni correction for multiple comparisons). The statistical tests were performed using R v3.4.3^76^.

## Supplementary information

Supplementary information

Supplementary data: video
