# Supplementary material for: Characterization of a large cluster of HIV-1 A1 infections detected in Portugal and connected to several Western European countries
Source: Sci Rep. 2019 May 10;9:7223. doi: 10.1038/s41598-019-43420-2 (PMC6510806; doi:10.1038/s41598-019-43420-2)
Supplement: Supplementary file 1 — Supplementary information [file 41598_2019_43420_MOESM1_ESM.docx]

**Characterization of a large cluster of HIV-1 A1 infections detected in Portugal and connected to several Western European countries**

Pedro M.M. Araújo^1,2^, Alexandre Carvalho^1,2,3^, Marta Pingarilho[^4^](https://www.ncbi.nlm.nih.gov/pubmed/?term=Pingarilho%20M%5BAuthor%5D&cauthor=true&cauthor_uid=29280759), BEST-HOPE study group^5^, Ana B. [Abecasis](https://www.ncbi.nlm.nih.gov/pubmed/?term=Abecasis%20AB%5BAuthor%5D&cauthor=true&cauthor_uid=29280759)^4^, Nuno S. Osório^1,2.*^

^1^ Life and Health Sciences Research Institute (ICVS), School of Medicine, University of Minho, Braga, Portugal

^2^ ICVS/3B’s - PT Government Associate Laboratory, Braga/Guimarães, Portugal;

^3^ Hospital de Braga, Braga, Portugal

^4^ Global Health and Tropical Medicine-GHTM, Institute for Hygiene and Tropical Medicine, Universidade NOVA de Lisboa, UNL, Lisbon, Portugal

^5^ A list of other contributors can be seen in the Acknowledgments section

* nosorio@med.uminho.pt

Supplementary Information

**Table 1:** Demographic data of the 65 sub-subtype A1 infected patients in the study

| **Gender** |  | N | Proportion |
| --- | --- | --- | --- |
|  | Male | 57 | 0.88 |
|  | Female | 8 | 0.12 |
|  | |  |  |
| **Birth country /Region** | |  |  |
|  | Portugal | 56 | 0.86 |
|  | Africa* | 4 | 0.06 |
|  | East Europe** | 4 | 0.06 |
|  | Brazil | 1 | 0.01 |
|  | |  |  |
| **Self-reported transmission route** | |  |  |
|  | Sexual | 65 | 1 |
|  | MSM | 37 | 0.57 |
|  | Heterosexual | 28 | 0.43 |
|  |  |  |  |
| **Age at Diagnosis** |  |  |  |
|  | <20 | 1 | 0.02 |
|  | ≥20 <35 | 33 | 0.51 |
|  | ≥35 <50 | 27 | 0.42 |
|  | <50 | 4 | 0.06 |

*Angola, Cape-Verde, Guiné-Bissau, Mozambique

**Ukraine, Russia

**Table 2**: Pairwise rates of diffusion between geographic locations (discreate traits).

| Locations | Bayes Factor | Posterior Probability |
| --- | --- | --- |
| Spain – Portugal | 5220.229 | 0.999 |
| Cyprus – Greece | 3683.501 | 0.999 |
| UK - Greece | 392.181 | 0.988 |
| Greece – USA | 210.814 | 0.978 |
| Spain – France | 17.974 | 0.795 |
| Belgium – Greece | 17.534 | 0.791 |
| Cyprus – Netherlands | 8.434 | 0.645 |
| Greece – Portugal | 5.57 | 0.545 |
| Greece – Kuwait | 5.261 | 0.531 |
| Greece – Sweden | 4.438 | 0.489 |
| Australia – USA | 4.324 | 0.482 |
| Spain – Greece | 3.968 | 0.461 |
| UK – Portugal | 3.778 | 0.449 |
| Australia – Greece | 3.464 | 0.427 |

**Table 3:** Protease sites with significant differences in the amino acid proportions. Correction of the p-value for 105 Fisher exact tests.

| Protein | Position ^a^ | Amino Acid | Percentage in MA1 group | Percentage in B group | Bonferroni corrected p |
| --- | --- | --- | --- | --- | --- |
| Protease | 12 | T | 2% (48/49) | 20% (25/41) | <0.001 |
|  | 14 ^b^ | R | 57% (24/42) | 10% (4/40) | <0.001 |
|  |  | K | 43% (18/42) | 90% (36/40) | <0.001 |
|  | 16 | G | 47% (22/47) | 98% (39/40) | <0.001 |
|  |  | E | 51% (24/47) | 3% (1/40) | <0.001 |
|  | 20 ^b^ | K | 47% (23/49) | 95% (37/39) | <0.001 |
|  |  | R | 53% (26/49) | 0% (0/39) | <0.001 |
|  | 35 ^b^ | E | 0% (0/49) | 56% (23/41) | <0.001 |
|  |  | D | 100% (49/49) | 44% (18/41) | <0.001 |
|  | 36 ^b, c^ | M | 0% (0/50) | 71% (29/41) | <0.001 |
|  |  | I | 100% (50/50) | 27% (11/41) | <0.001 |
|  | 37 ^b^ | N | 100% (50/50) | 24% (8/34) | <0.001 |
|  |  | E | 0% (0/50) | 29% (10/34) | 0.005 |
|  |  | D | 0% (0/50) | 24% (8/34) | 0.044 |
|  | 41 ^b^ | R | 0% (0/50) | 72% (28/39) | <0.001 |
|  |  | K | 100% (50/50) | 28% (11/39) | <0.001 |
|  | 45 | K | 2% (1/48) | 97% (38/39) | <0.001 |
|  |  | R | 98% (47/48) | 3% (1/39) | <0.001 |
|  | 57 ^b^ | R | 8% (4/49) | 46% (18/39) | 0.006 |
|  |  | K | 92% (45/49) | 54% (18/39) | 0.006 |
|  | 63 ^b, d^ | A | 48% (22/46) | 8% (3/38) | 0.008 |
|  |  | P | 0% (0/46) | 47% (18/38) | <0.001 |
|  |  | T | 50% (23/46) | 13% (5/38) | 0.045 |
|  | 64 ^b, e^ | I | 100% (50/50) | 78% (32/41) | 0.047 |
|  | 69 | H | 0% (0/50) | 100% (42/42) | <0.001 |
|  |  | K | 100% (50/50) | 0% (0/42) | <0.001 |
|  | 71 ^b^ | A | 98% (48/49) | 72% (28/39) | 0.044 |
|  | 89 ^b^ | M | 96% (48/50) | 7% (3/41) | <0.001 |
|  |  | L | 0% (0/50) | 93% (38/41) | <0.001 |

^a^ Amino acid position based on the reference sequence HXB2(GenBank: K03455.1);

^b^ Position was previously reported by Parera *et. al* ^1^ as impactful in the protease catalytic efficiency;

^c^ Position reported by Costa M. *et. al* ^2^^2^ with amino acid variants impacting the viral maturation;

^d^ Position was previously associated with replication fitness compensatory mutations by Suñé C. *et. al* ^3^;

^e^ Position associated with differences in the viral replication capacity by Ng O. *et. al* ^4^.

| Protein | Position ^a^ | Amino Acid | | Percentage in MA1 group | Percentage in B group | Bonferroni corrected p |
| --- | --- | --- | --- | --- | --- | --- |
| Reverse Transcriptase | 35 | V | 0% (0/29) | | 71% (5/7) | 0.013 |
|  |  | T | 100% (29/29) | | 0% (0/7) | <0.001 |
|  | 40 | E | 13% (6/46) | | 100% (42/42) | <0.001 |
|  |  | D | 87% (40/46) | | 0% (0/42) | <0.001 |
|  | 49 | K | 13% (6/47) | | 95% (38/40) | <0.001 |
|  |  | R | 87% (41/47) | | 5% (2/40) | <0.001 |
|  | 60 | V | 0% (0/50) | | 83% (35/42) | <0.001 |
|  |  | I | 100% (50/50) | | 17% (7/42) | <0.001 |
|  | 122 | E | 85% (41/48) | | 20% (8/40) | <0.001 |
|  |  | K | 8% (4/48) | | 78% (31/40) | <0.001 |
|  | 123 | N | 44% (19/43) | | 3% (1/37) | 0.003 |
|  | 162 | S | 98% (48/49) | | 64% (27/42) | 0.008 |
|  | 169 | D | 48% (23/48) | | 8% (3/39) | 0.001 |
|  |  | E | 52% (25/48) | | 92% (36/39) | 0.001 |
|  | 173 | S | 51% (21/41) | | 0% (0/41) | <0.001 |
|  |  | K | 0% (0/41) | | 93% (38/41) | <0.001 |
|  |  | L | 29% (12/41) | | 0% (0/41) | 0.046 |
|  | 174 | Q | 0% (0/47) | | 100% (41/41) | <0.001 |
|  |  | K | 100% (47/47) | | 0% (0/41) | <0.001 |
|  | 179 | V | 6% (3/47) | | 95% (39/41) | <0.001 |
|  |  | I | 94% (44/47) | | 5% (2/41) | <0.001 |
|  | 200 | A | 63% (29/46) | | 9% (3/35) | <0.001 |
|  |  | T | 26% (12/46) | | 74% (26/35) | 0.005 |
|  | 207 ^b^ | A | 94% (46/49) | | 5% (2/38) | <0.001 |
|  |  | Q | 0% (0/49) | | 74% (28/38) | <0.001 |
|  | 211 | S | 90% (44/49) | | 0% (0/35) | <0.001 |
|  |  | K | 4% (2/49) | | 69% (24/35) | <0.001 |
|  | 245 | M | 37% (18/49) | | 0% (0/40) | 0.001 |
|  | 250 | D | 10% (3/29) | | 88% (7/8) | 0.020 |
|  |  | E | 90% (26/29) | | 12% (1/8) | 0.020 |
|  | 286 | A | 100% (27/27) | | 37% (3/8) | 0.042 |
|  |  | T | 0% (0/27) | | 63% (5/8) | 0.042 |
|  | 291 | E | 0% (0/27) | | 88% (7/8) | <0.001 |
|  |  | D | 100% (27/27) | | 13% (1/8) | <0.001 |
|  | 335 | G | 14% (4/28) | | 100% (8/8) | 0.004 |
|  |  | D | 86% (24/28) | | 0% (0/8) | 0.004 |

**Table 4:** Reverse transcriptase sites with significant differences in the amino acid proportions. Correction of the p-value for 241 Fisher exact tests.

^a^ Amino acid position based on the reference sequence HXB2(GenBank: K03455.1);

^b^ Position in the reverse transcriptase associated with viral fitness alterations by Lu J. *et. Al* ^5^.

**Table 5:** List of sites with amino acid variants associated with differences in the CD4^+^ T cell counts. Correction of the p-value for 48 comparisons in the protease and 78 in the reverse transcriptase coding regions.

|  | Comparison | p-value | Bonferroni corrected p |
| --- | --- | --- | --- |
| PR | R14 VS K14 | 0.05 ^a^ | 2.34 |
|  | I36 VS M36 | 0.04 ^a^ | 1.96 |
|  | M36 VS others | 0.03 ^a^ | 1.59 |
|  | R45 VS K45 | 0.02 ^a^ | 1.07 |
|  | H69 VS K69 | 0.03 ^a^ | 1.22 |
|  | T71 VS others | 0.05 ^a^ | 2.18 |
|  | M89 VS others | 0.02 ^a^ | 1.06 |
|  | L89 VS M89 | 0.01 ^b^ | 0.69 |
|  | L89 VS others | 0.01 ^b^ | 0.58 |
| RT | T35 VS others | 0.05 ^a^ | 3.85 |
|  | E40 VS D40 | 0.03 ^a^ | 2.66 |
|  | L173 VS K173 | 0.03 ^a^ | 1.98 |
|  | K173 VS others | 0.03 ^a^ | 2.62 |
|  | K174 VS Q174 | 0.03 ^a^ | 2.28 |
|  | Q207 VS A207 | 0.04 ^a^ | 3.26 |
|  | A207 VS others | 0.04 ^a^ | 3.32 |

^a^ Welch T-test; ^b^ Mann-Whitney-Wilcoxon Test.

**Table 6:** List of sites with amino acid variants associated with differences in the viral load (logarithm). Correction of the p-value for 48 comparisons in the protease and 78 in the reverse transcriptase coding regions.

|  | Comparison | p-value | Bonferroni corrected p |
| --- | --- | --- | --- |
| PR | R14 VS K14 | 0.01 ^a^ | 0.45 |
|  | I15 VS V15 | 0.04 ^a^ | 1.95 |
|  | R45 VS K45 | 0.04 ^a^ | 1.83 |
|  | H69 VS K69 | 0.02 ^b^ | 1.00 |
|  | L89 VS others | 0.04 ^b^ | 1.97 |
| RT | T35 VS others | 0.02 ^b^ | 1.20 |
|  | T35 VS V35 | 0.05 ^b^ | 3.57 |
|  | V35 VS others | 0.04 ^a^ | 2.97 |
|  | E40 VS D40 | 0.02 ^a^ | 1.86 |
|  | R49 VS K49 | 0.02 ^a^ | 1.64 |
|  | K174 VS Q174 | 0.03 ^b^ | 1.98 |
|  | E177 VS D177 | 0.05 ^a^ | 3.67 |
|  | D177 VS others | 0.03 ^a^ | 2.18 |
|  | I179 VS V179 | 0.01 ^b^ | 0.98 |
|  | Q207 VS A207 | 0.04 ^b^ | 3.08 |
|  | A207 VS others | 0.01 ^b^ | 1.15 |
|  | P272 VS others | 0.03 ^a^ | 2.16 |
|  | K275 VS Q275 | 0.02 ^b^ | 1.37 |
|  | I293 VS V293 | 0.02 ^a^ | 1.20 |
|  | P294 VS T294 | 0.04 ^b^ | 3.26 |
|  | P294 VS others | 0.04 ^b^ | 2.79 |

^a^ Welch T-test; ^b^ Mann-Whitney-Wilcoxon Test.

**Table 7:** Distribution of the study 65 A1 sub-subtype samples by hospital where they were obtained.

| Hospital | Region | Number of patients infected with A1 | Sampling dates (from - to) | Portuguese | Non-Portuguese |
| --- | --- | --- | --- | --- | --- |
| CHA - Faro | South | 2 | 2016 – 2017 | 2 | 0 |
| CHA - Portimão | South | 1 | 2016 – 2017 | 0 | 1 |
| CHBV - Aveiro | Center | 3 | 2016 – 2017 | 3 | 0 |
| CHP - Porto | North | 2 | 2016 – 2017 | 2 | 0 |
| São Bernardo-Setúbal | Center | 2 | 2016 – 2017 | 2 | 0 |
| CH S. João -  Porto | North | 7 | 2016 – 2017 | 7 | 0 |
| CHUC - Coimbra | Center | 3 | 2016 – 2017 | 3 | 0 |
| CHLO - Egas Moniz | Center | 1 | 2016 – 2017 | 0 | 1 |
| Beatriz-Ângelo - Loures | Center | 2 | 2016 – 2017 | 1 | 1 |
| CHLC- Curry Cabral Lisboa | Center | 1 | 2016 – 2017 | 1 | 0 |
| Garcia da Orta -  Almada | Center | 2 | 2016 – 2017 | 1 | 1 |
| CHLC- Capuchos Lisboa | Center | 2 | 2016 – 2017 | 2 | 0 |
| CHLC- São José Lisboa | Center | 1 | 2016 – 2017 | 1 | 0 |
| HB - Braga | North | 36 | 2006 - 2017 | 31 | 5 |

**Table 8:** List of the Genbank IDs corresponding to the study sequences separated by subtype

| B subtype (N=42) |
| --- |
| KM205983.1, KM206066.1, KM206067.1, KM206098.1, KM206061.1, KM205834.1, KM205940.1, KM205910.1, KM206056.1, KM206021.1, KM206050.1, KM206073.1,KM206095.1, KM205891.1, KM206007.1, KM206072.1, KM205890.1, KM205936.1, KM206052.1, KM205926.1, KM205869.1, KM205998.1, KM205934.1, KM205955.1, KM206026.1, KM205884.1, KM206069.1, KM205972.1, KM205947.1, MK766235.1, MK766236.1, MK766237.1, MK766238.1, MK766239.1, MK766240.1, MK766241.1, MK766242.1, MK766243.1, MK766244.1, MK766245.1, MK766246.1, MK766247.1 |
| A1 sub-subtype (N=36) * |
| KM205930.1, KM205896.1, KM206012.1, KM205994.1, KM205969.1, KM206004.1, KM205857.1, KM205957.1, KM205965.1, KM205911.1, KM205942.1, KM205892.1, KM205852.1, MG807846, MG807847, MK766248.1, MK766249.1, MK766250.1, MK766251.1, MK766252.1, MK766253.1, MK766254.1, MK766255.1, MK766256.1, MK766257.1, MK766258.1, MK766259.1, MK766260.1, MK766261.1, MK766262.1, MK766263.1, MK766264.1, MK766265.1, MK766266.1, MK766267.1, MK766268.1 |

*The remaining A1 sub-subtype sequences (N=29) are in the BEST-HOPE program database and can be obtained upon request to: besthope.ihmt@gmail.com;

**Table 9:** Bayesian analysis ^6-8^ model evaluation. Different combinations of coalescent tree priors (constant, exponential, skyline ^9^, and skygrid ^1^^0^) and clock methods (strict, and relaxed log normal^1^^1^) were compared using path sampling (PS) and stepping-stone (SS) ^1^^2^^-^^1^^4^ sampling. For the run of each set of parameters there was an initial burn-in run of 6 million states, followed by 100 or 150 steps of 300 thousand iterations to estimate PS and SS. From the comparison between the 100 and 150 sampling steps it was possible to infer the stabilization of the estimation. These comparisons highlighted the coalescent skygrid model with an uncorrelated relaxed clock has the best fitting model.

| Number of steps | Site model | Clock model | Coalescent tree model | PS | SS | Rank |
| --- | --- | --- | --- | --- | --- | --- |
| 150 | GTR +G4 +I | urcln | skygrid | -6526.09 | -6528.02 | 1 |
|  | GTR +G4 +I | urcln | skyline | -6542.91 | -6544.13 | 2 |
|  | GTR +G4 +I | strick | skygrid | -6545.67 | -6547.72 | 3 |
|  | GTR +G4 +I | strick | skyline | -6552.33 | -6553.98 | 4 |
|  | GTR +G4 +I | urcln | exponential | -6555.02 | -6555.91 | 5 |
|  | GTR +G4 +I | strick | exponential | -6559.18 | -6560.01 | 6 |
|  | GTR +G4 +I | urcln | constant | -6560.70 | -6561.77 | 7 |
|  | GTR +G4 +I | strick | constant | -6567.12 | -6568.45 | 8 |
| 100 | GTR +G4 +I | urcln | skygrid | -6529.31 | -6532.41 | 1 |
|  | GTR +G4 +I | urcln | skyline | -6544.34 | -6546.49 | 2 |
|  | GTR +G4 +I | strick | skygrid | -6549.69 | -6551.25 | 3 |
|  | GTR +G4 +I | urcln | exponential | -6551.60 | -6552.66 | 4 |
|  | GTR +G4 +I | strick | skyline | -6555.56 | -6557.89 | 5 |
|  | GTR +G4 +I | strick | exponential | -6556.41 | -6558.38 | 6 |
|  | GTR +G4 +I | urcln | constant | -6559.21 | -6561.63 | 7 |
|  | GTR +G4 +I | strick | constant | -6562.51 | -6564.16 | 8 |

**Table 10:** Permutations of criteria for transmission cluster support. The tested values for likelihood ratio test (aLRT) SH-like branch support (estimated with PhyML v3) were 0.90 and 0.95. For the branch posterior probability support (estimated with BEAST v1.8) the values 0.95 and 0.99 were evaluated. For cluster genetic distance the values 0.03 and 0.025 substitutions per site where tested for the mean while for the maximin the values 0.05 and 0.045 were evaluated. The combination number 01 was selected since it achieved a relative high number of cluster (8) using strict parameters.

| Combination number | aLRT ≥ | Posterior probability ≥ | Median genetic distance ≤ | Maximum genetic distance ≤ | Number of clusters |
| --- | --- | --- | --- | --- | --- |
| 01 | 0.95 | 0.99 | 0.03 | 0.05 | 8 |
| 02 | 0.95 | 0.99 | 0.03 | 0.045 | 7 |
| 03 | 0.95 | 0.99 | 0.025 | 0.05 | 6 |
| 04 | 0.95 | 0.99 | 0.025 | 0.045 | 6 |
| 05 | 0.95 | 0.95 | 0.03 | 0.05 | 8 |
| 06 | 0.95 | 0.95 | 0.025 | 0.05 | 6 |
| 07 | 0.95 | 0.95 | 0.025 | 0.045 | 6 |
| 08 | 0.95 | 0.95 | 0.03 | 0.045 | 7 |
| 09 | 0.9 | 0.99 | 0.03 | 0.05 | 8 |
| 10 | 0.9 | 0.99 | 0.03 | 0.045 | 7 |
| 11 | 0.9 | 0.99 | 0.025 | 0.05 | 6 |
| 12 | 0.9 | 0.99 | 0.025 | 0.045 | 6 |
| 13 | 0.9 | 0.95 | 0.03 | 0.05 | 8 |
| 14 | 0.9 | 0.95 | 0.025 | 0.05 | 6 |
| 15 | 0.9 | 0.95 | 0.025 | 0.045 | 6 |
| 16 | 0.9 | 0.95 | 0.03 | 0.045 | 7 |

**Table 11**: Descriptive statistics and distribution of the sub-subtype A1 and B groups for Age, proportion of ambiguous sites (PAS), viral load, and CD4+ T cell counts. Data regarding the study MA1 group, matched subtype B group, and all the available subtype B samples in the cohort.

|  | Group | Sub-subtype A1 | All subtype B | Matched subtype B |
| --- | --- | --- | --- | --- |
|  | **Number** | 50 | 170 | 42 |
| Age | **Mean** | 33.86 | - | 34.21 |
|  | **Std. Dev.** | 9.47 | - | 10.04 |
| PAS | **Mean** | 0.0073 | - | 0.0089 |
|  | **Std. Dev.** | 0.0077 | - | 0.0056 |
| Viral load (log10) | **Mean** | 4.56 | 4.82 | 4.91 |
|  | **Std. Dev.** | 0.85 | 0.86 | 0.84 |
| CD4+ count | **Mean** | 426.72 | 356.61 | 325.81 |
|  | **Std. Dev.** | 195.92 | 250.04 | 219.91 |
|  | **< 200 (N)** | 6 | 51 | 15 |
|  | **≥200 < 500 (N)** | 26 | 79 | 18 |
|  | **≥ 500(N)** | 18 | 40 | 9 |

‘-’: Not calculated due to missing data

**A**


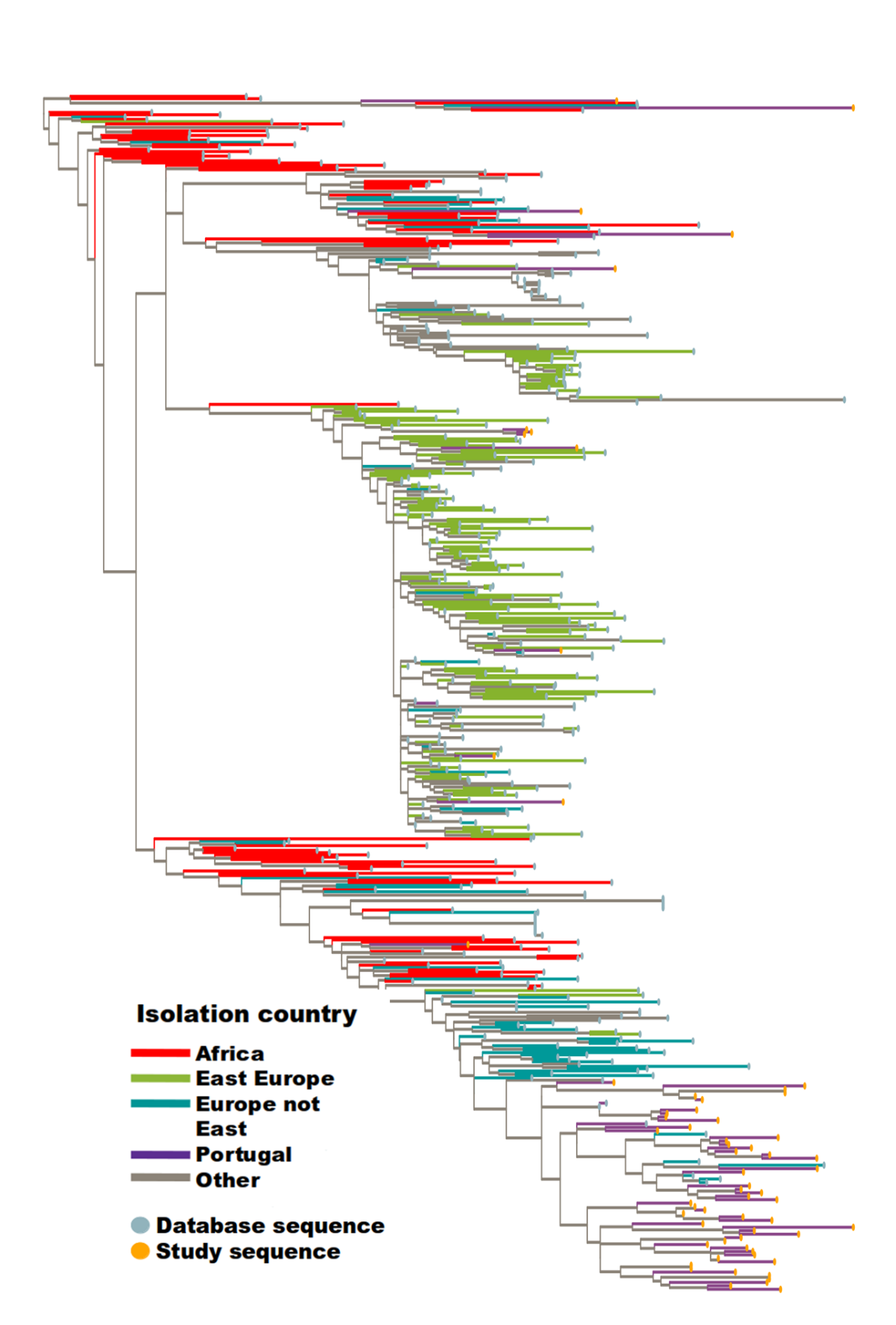


**Figure 1: Phylogenetic representation of the A1 sequences isolated in Portugal and closely related sequences from databases**. Maximum likelihood tree with the study A1 sequences and the closely related database sequences (n=490). Branch colours indicate the geographical origin of the sequences. The color in the tip points indicates the sequence was obtained in this study (yellow) or from databases (grey).

**Figure 2: Maximum likelihood phylogenetic reconstructions of the subset of 99 taxa more closely related with the MA1 sequences (manuscript Figure 2).** A: Gag and Pol genomic regions used; B: Gag, Pol, and Env genomic regions used.
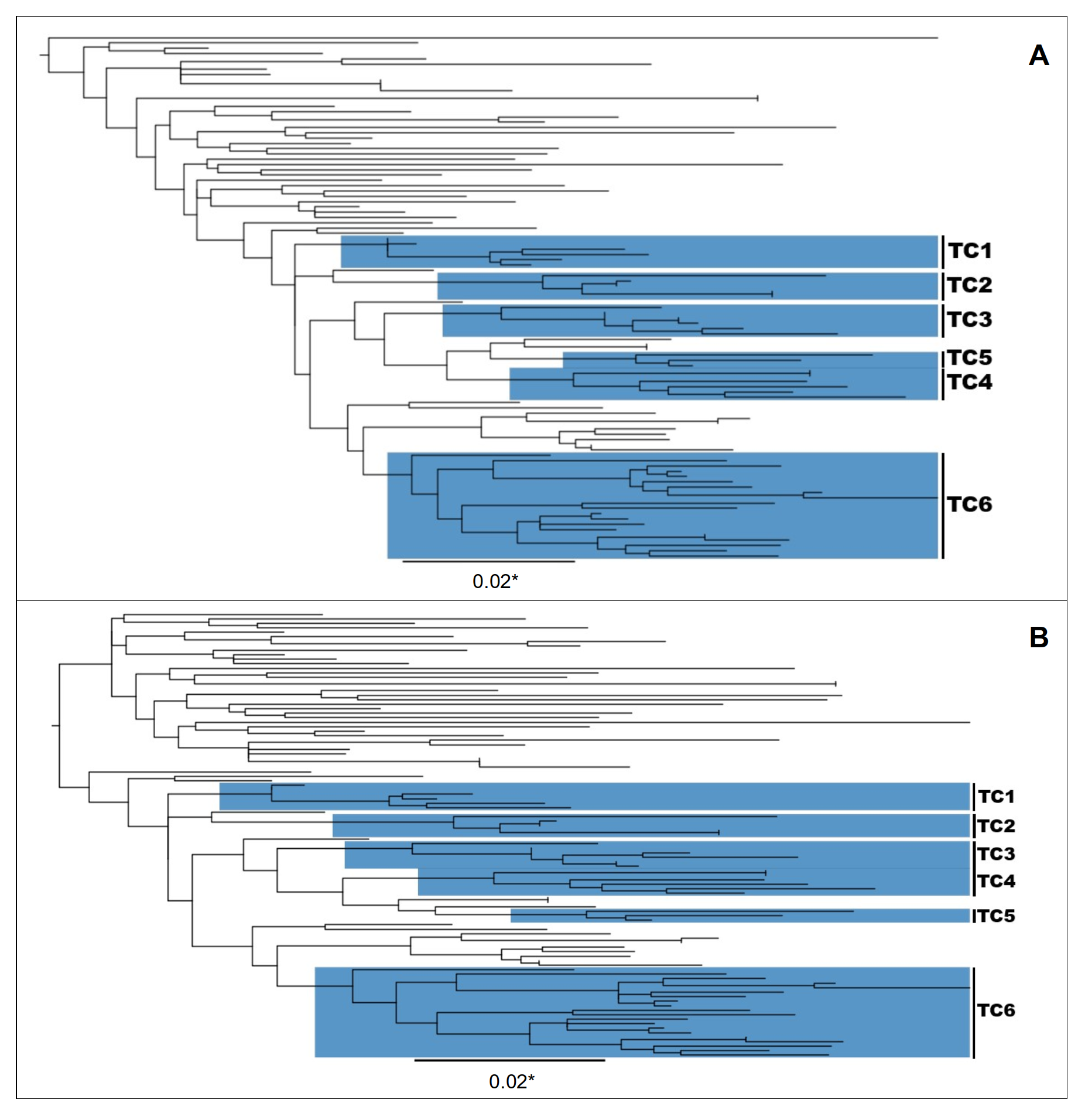
* Genetic distance in mutations per site.


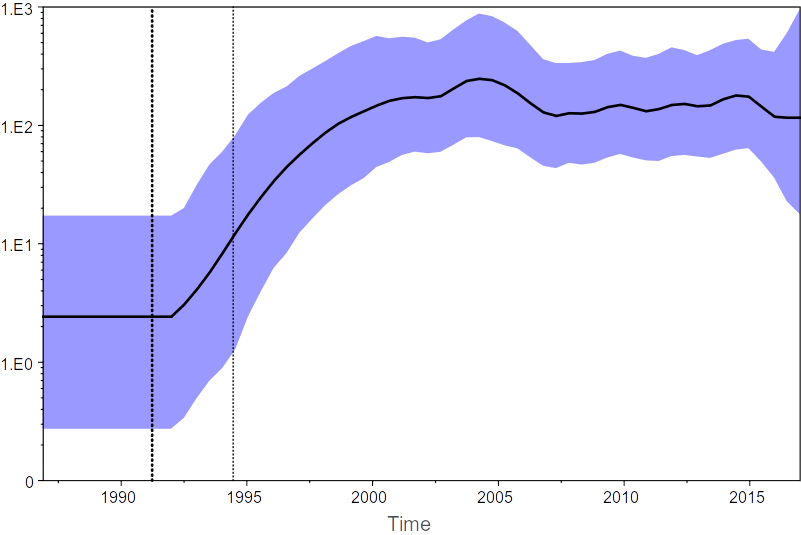


**Figure 3: Skygrid plot inferred with BEAST 1.8.4 using of the subset of 99 taxa more closely related with the MA1 sequences (manuscript Figure 2).**

**References**

1. Parera, M., Fernandez, G., Clotet, B. & Martinez, M. A. HIV-1 Protease Catalytic Efficiency Effects Caused by Random Single Amino Acid Substitutions. *Mol. Biol. Evol.* **24,** 382–387 (2006).

2. Costa, M. G. S. *et al.* Impact of M36I polymorphism on the interaction of HIV-1 protease with its substrates: insights from molecular dynamics. *BMC Genomics* **15 Suppl 7,** S5 (2014).

3. Suñé, C., Brennan, L., Stover, D. R. & Klimkait, T. Effect of polymorphisms on the replicative capacity of protease inhibitor-resistant HIV-1 variants under drug pressure. *Clin. Microbiol. Infect.* **10,** 119–26 (2004).

4. Ng, O. T. *et al.* HIV type 1 polymerase gene polymorphisms are associated with phenotypic differences in replication capacity and disease progression. *J. Infect. Dis.* **209,** 66–73 (2014).

5. Lu, J., Whitcomb, J. & Kuritzkes, D. R. Effect of the Q207D mutation in HIV type 1 reverse transcriptase on zidovudine susceptibility and replicative fitness. *J. Acquir. Immune Defic. Syndr.* **40,** 20–3 (2005).

6. Drummond, A. J., Suchard, M. a., Xie, D. & Rambaut, A. Bayesian phylogenetics with BEAUti and the BEAST 1.7. *Mol. Biol. Evol.* **29,** 1969–1973 (2012).

7. Ferreira, M. A. R. & Suchard, M. A. Bayesian analysis of elapsed times in continuous-time Markov chains. *Can. J. Stat.* **36,** 355–368 (2008).

8. Ayres, D. L. *et al.* BEAGLE: an application programming interface and high-performance computing library for statistical phylogenetics. *Syst. Biol.* **61,** 170–173 (2012).

9. Drummond, A. J., Rambaut, A., Shapiro, B. & Pybus, O. G. Bayesian coalescent inference of past population dynamics from molecular sequences. *Mol. Biol. Evol.* **22,** 1185–1192 (2005).

10. Gill, M. S. *et al.* Improving bayesian population dynamics inference: A coalescent-based model for multiple loci. *Mol. Biol. Evol.* **30,** 713–724 (2013).

11. Drummond, A. J., Ho, S. Y. W., Phillips, M. J. & Rambaut, A. Relaxed phylogenetics and dating with confidence. *PLoS Biol.* **4,** 699–710 (2006).

12. Baele, G. *et al.* Improving the accuracy of demographic and molecular clock model comparison while accommodating phylogenetic uncertainty. *Mol. Biol. Evol.* **29,** 2157–2167 (2012).

13. Baele, G., Li, W. L. S., Drummond, A. J., Suchard, M. A. & Lemey, P. Accurate model selection of relaxed molecular clocks in Bayesian phylogenetics. *Mol. Biol. Evol.* 30, 239–243 (2013).

14. Baele, G., Lemey, P. & Suchard, M. A. Genealogical Working Distributions for Bayesian Model Testing with Phylogenetic Uncertainty. *Syst. Biol.* 65, 250–264 (2016).
